# Dissecting a diagnostic enigma: Hypertension in a young patient from an organ of Zuckerkandl paraganglioma

**DOI:** 10.1002/ccr3.8061

**Published:** 2023-10-17

**Authors:** Reabal Najjar, Elizabeth Paver, Jonathan McGuane, Gert Frahm‐Jensen

**Affiliations:** ^1^ The Australian National University Medical School, College of Health and Medicine, Australian Capital Territory Canberra Australian Capital Territory Australia; ^2^ The Canberra Hospital, Canberra Health Services Canberra Australian Capital Territory Australia; ^3^ ACT Pathology, The Canberra Hospital Canberra Australian Capital Territory Australia; ^4^ Department of Vascular Surgery The Canberra Hospital Canberra Australian Capital Territory Australia

**Keywords:** organ of Zuckerkandl, paraganglioma, pathology, radiology, vascular surgery

## Abstract

Hypertension in young patients can mask rare conditions like paragangliomas, especially in the absence of conventional symptoms. A comprehensive diagnostic evaluation and multidisciplinary approach are crucial for optimal management and outcomes.

## Graphical abstract

Contrast‐enhanced computed tomography showcasing a distinct lesion near the aortic bifurcation, consistent with a paraganglioma arising from the Organ of Zuckerkandl. This report delves into the challenges in diagnosing and managing a rare presentation of a functional paraganglioma in a 28‐year‐old male with resistant hypertension.

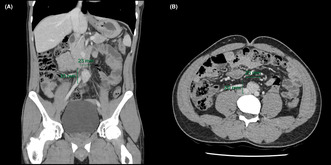

## 1 INTRODUCTION

Resistant labile hypertension, often indicative of underlying pathologies, can be a perplexing clinical entity to diagnose and manage, with adrenal neuroendocrine paragangliomas being a notable culprit. We present a unique case of a young male whose sole presentation was resistant hypertension, lacking classic symptoms of catecholamine excess, traced to a paraganglioma in the Organ of Zuckerkandl. Emphasizing the diagnostic and therapeutic challenges, particularly when conventional antihypertensive therapies prove ineffective, this report advocates for an integrative diagnostic strategy and multidisciplinary treatment approach. By shedding light on this diagnostic labyrinth, we aim to provide a nuanced understanding of paragangliomas and offer insights to clinicians navigating similar cases.

## 2 CASE PRESENTATION

A 33‐year‐old male presented to his general practitioner (GP) with blurred vision and eye discomfort over 2 weeks. His visual acuity was found to be 6/6 bilaterally without additional ocular symptoms. A thorough physical examination showed persistently elevated blood pressure, ranging from 150/90 mmHg to 180/100 mmHg, in the absence of any other abnormal findings. The patient denied experiencing any characteristic hypertensive symptoms, such as palpitations, chest pain, dyspnoea, headaches, dizziness, nausea, vomiting, flushing, diaphoresis, insomnia, or anxiety. The patient had no notable personal, medical, or family history, led a healthy diet, and had a body mass index of 20.6 kg/m^2^. Initial antihypertensive therapy using prazosin was ineffective, even after dosage adjustment.

## 3 INVESTIGATIONS

Exhaustive investigations into the secondary causes of hypertension mostly yielded results within the normal range with one standout exception: markedly elevated serum normetanephrine levels, measured at 4550 pmol/L. Other indices, including metanephrine, 3‐Methoxytyramine, renal function, the renin‐angiotensin‐aldosterone system, endocrine studies, and thyroid function, were within standard thresholds (Table 1).

**TABLE 1:** Summary of the patient's initial serum investigations.

Parameter	Result	Reference range
Normetanephrine	4550 ↑	<610 pmol/L
Metanephrine	330	<447 pmol/L
3‐Methoxytyramine	80	<181 pmol/L
Creatinine	65	60–110 umol/L
eGFR	>90	>90 mL/min/1.73 m^2^
Aldosterone	269	100–950 pmol/L
Renin	20	3.3–41 mU/L
Aldosterone/Renin ratio	13	<70
Cortisol (fasting)	271	100–535 nmol/L
TSH	3.4	0.40–3.5 mLU/L

**FIGURE 1:**
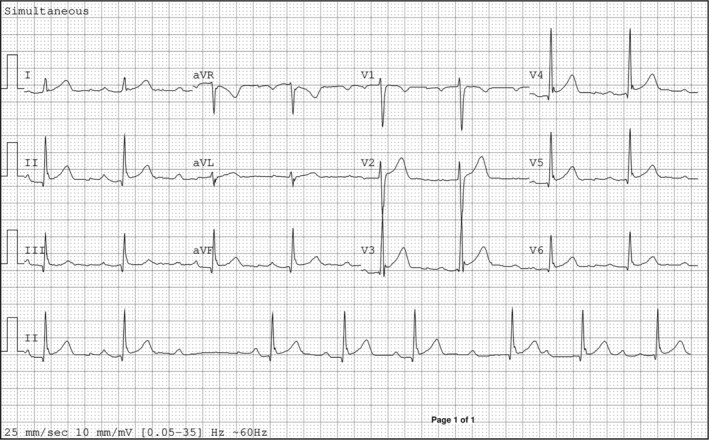
ECG image demonstrating a sinus rhythm with second degree Mobitz Type I AV block.65 BPM, QRS axis 67°, T axis 46°, RR interval 1131 ms, QRS duration 88 ms, QT interval 404 ms, corrected QT 381 ms.

24‐h ambulatory blood pressure monitoring indicated average systolic readings of 136 mmHg (day) and 126 mmHg (night), with a peak of 171/96 mmHg (day) and 146/77 mmHg (night). Systolic dipping was significantly reduced, observed at just 7.4%. The electrocardiogram showed a sinus rhythm with intermittent episodes of 2nd degree Mobitz type I (Wenckebach) atrioventricular blocks, but otherwise normal electrical activity and intervals (Figure 1).

**FIGURE 2:**
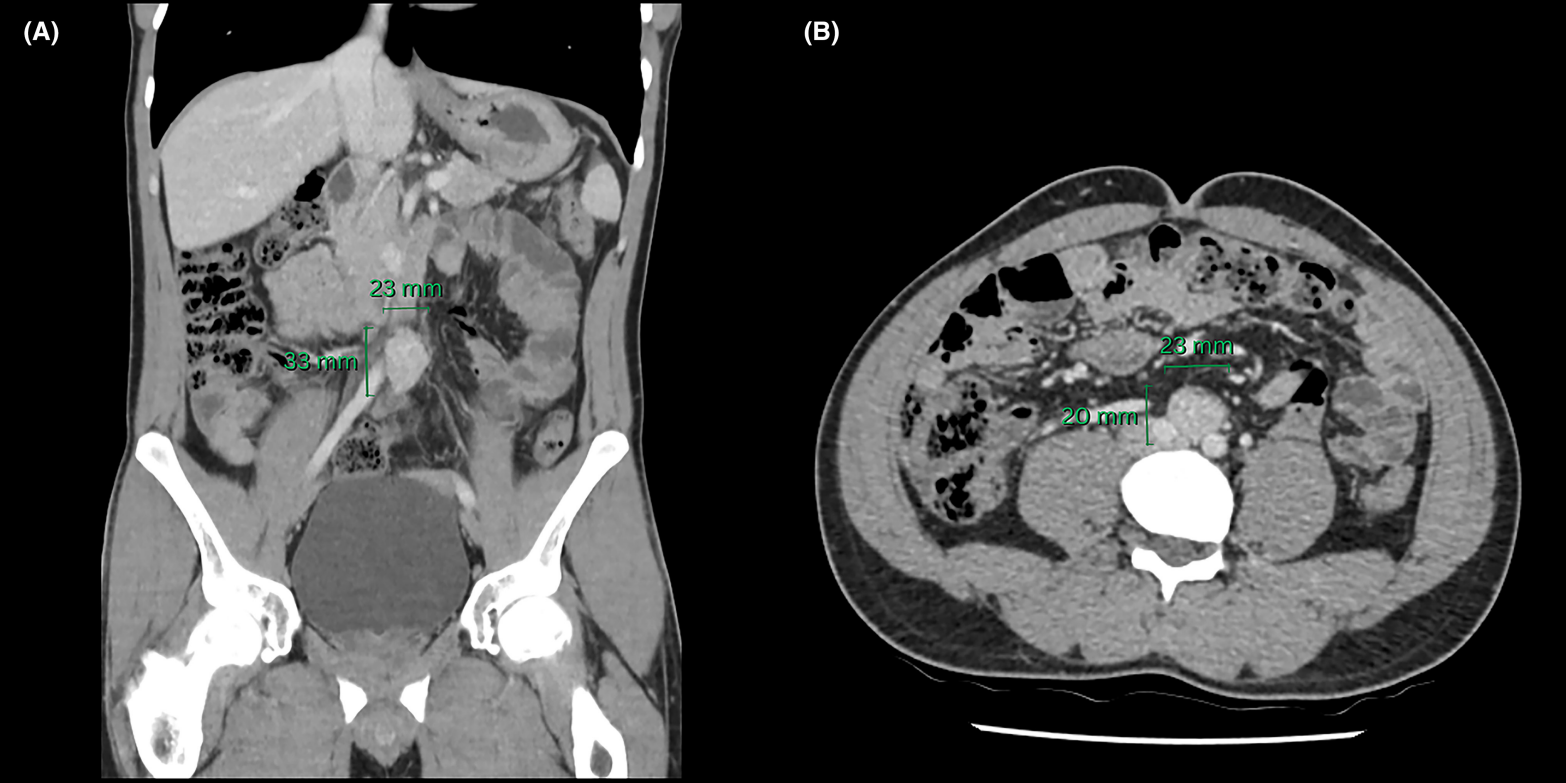
Coronal (A) and axial (B) abdominal CT slices depicting the 33 × 23 × 20 mm enhancing mass near the aortic bifurcation.

**FIGURE 3:**
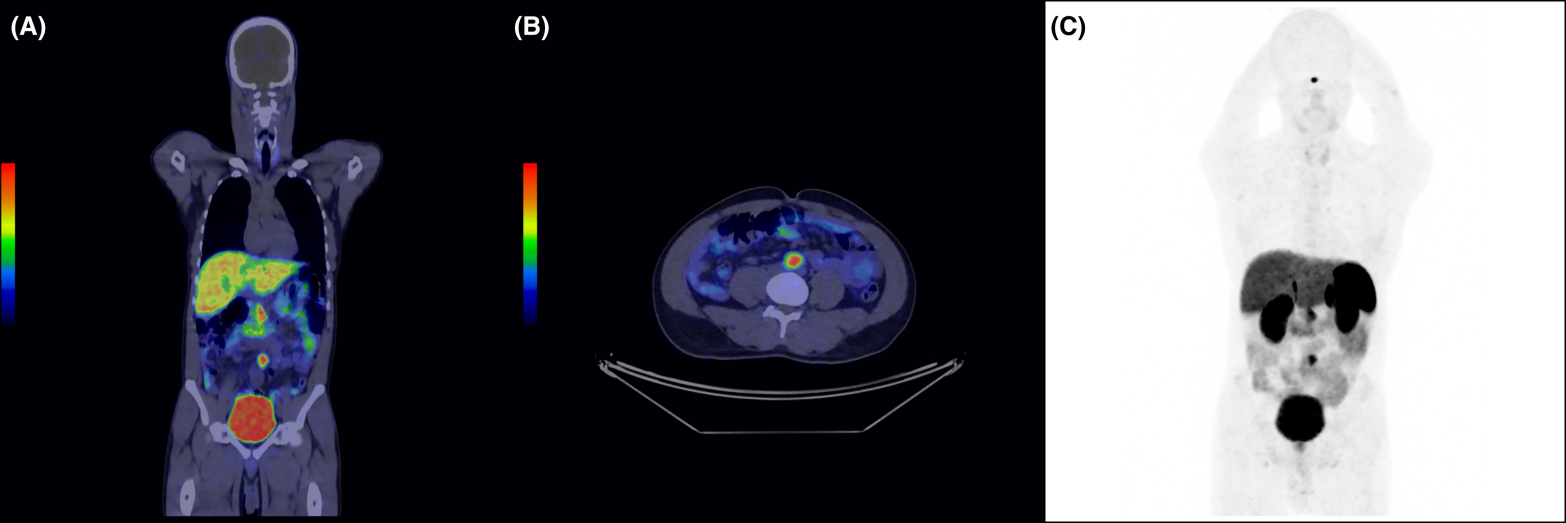
Coronal (A), axial (B), and MIP (C) views of the DOTATATE PET, showing significant radiotracer uptake (SUVmax 10.8) within the 23 × 33 mm mass located at the aortic bifurcation. MIP: Maximum intensity projection, PET: positron emission tomography, SUVmax: maximum standardized uptake value.

A contrast‐enhanced CT of the abdomen and pelvis revealed a 33 × 23 × 20 mm ellipsoid mass near the aortic bifurcation, strongly suggestive of an Organ of Zuckerkandl paraganglioma (Figure 2). A subsequent 68Ga DOTATATE PET/CT scan corroborated this suspicion, demonstrating intense radiotracer uptake within the lesion, with no abnormal tracer uptake observed elsewhere (Figure 3).

**FIGURE 4:**
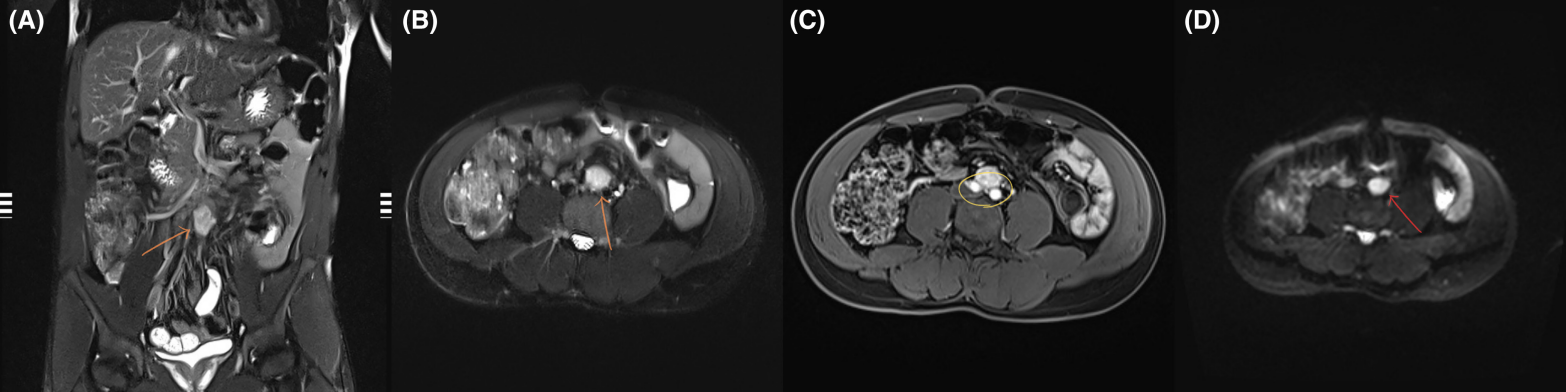
Coronal T2 fat‐saturated (A), axial T2 fat‐saturated (B), gadolinium‐enhanced axial T1 fat‐saturated (C), and DWI (D) MRI images showcasing the 21 × 21 × 29 mm lesion at the aortic bifurcation. The lesion demonstrates increased T2 signal (orange arrow), heterogeneous enhancement patterns (yellow circle), and restricted diffusion (red arrow).

An abdominal MRI report initially indicated the mass as an enlarged necrotic lymph node due to its ambiguous appearance. However, a subsequent evaluation by a different radiologist accurately identified it as a paraganglioma due to its increased T2 signal, restricted diffusion, heterogeneous enhancement patterns, and absence of fluid suggestive of necrosis. The lesion was located near the medial walls of the common iliac arteries and the anterior aspect of the left common iliac vein (Figure 4).

## 4 SURGICAL EXCISION

Upon confirming the diagnosis of an organ of Zuckerkandl paraganglioma through biochemical and radiological tests, the patient was promptly referred to the vascular surgery outpatient clinic. After discussing the procedure, risks, and anticipated outcomes, informed consent was obtained for open tumor excision. Six weeks postconsultation, the patient underwent a para‐umbilical midline laparotomy to remove the tumor at the aortic bifurcation. The complexity of the operation necessitated meticulous preoperative planning due to the risk of a catecholamine‐induced hypertensive crisis by pre‐emptively adjusting the dosage of calcium channel blockers.

The procedure, lasting approximately 5 h, involved the excision of the Organ of Zuckerkandl mass found adhering to the left common iliac vein necessitating a partial venous resection. For the repair, a bovine pericardium patch was chosen due to its biocompatibility, durability, and low thrombogenicity, ensuring a secure and long‐lasting repair. The surgery led to an estimated blood loss of 250 mL, which was recirculated to the patient through a cell saver.

**FIGURE 5:**
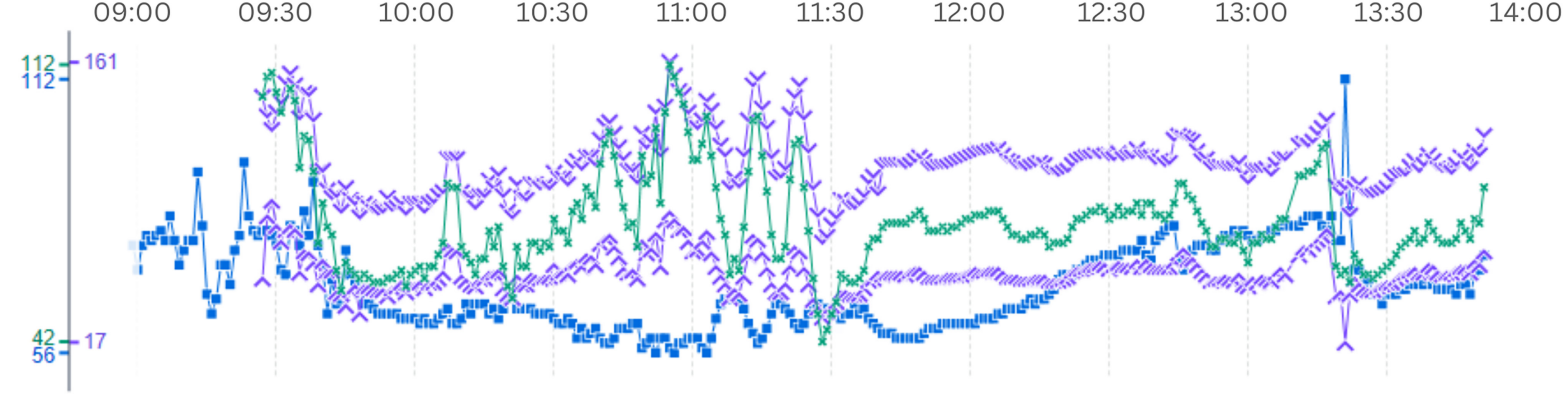
Graphical depiction of intraoperative vital signs, displaying significant systolic blood pressure spikes during manual tumor manipulation, peaking at 161/84.Blood pressure, mmHg (Purple); mean blood pressure, mmHg (green); pulse, BPM (blue).

Throughout the procedure, the patient's blood pressure exhibited fluctuations incited by the physiological response to the catecholamine release during tumor manipulation. This was effectively managed intraoperatively by titrating the administration of Clevidipine, Dexmedetomidine, and Norepinephrine, ensuring that the patient's systolic pressure remained within a safe limit (Figure 5).

**FIGURE 6:**
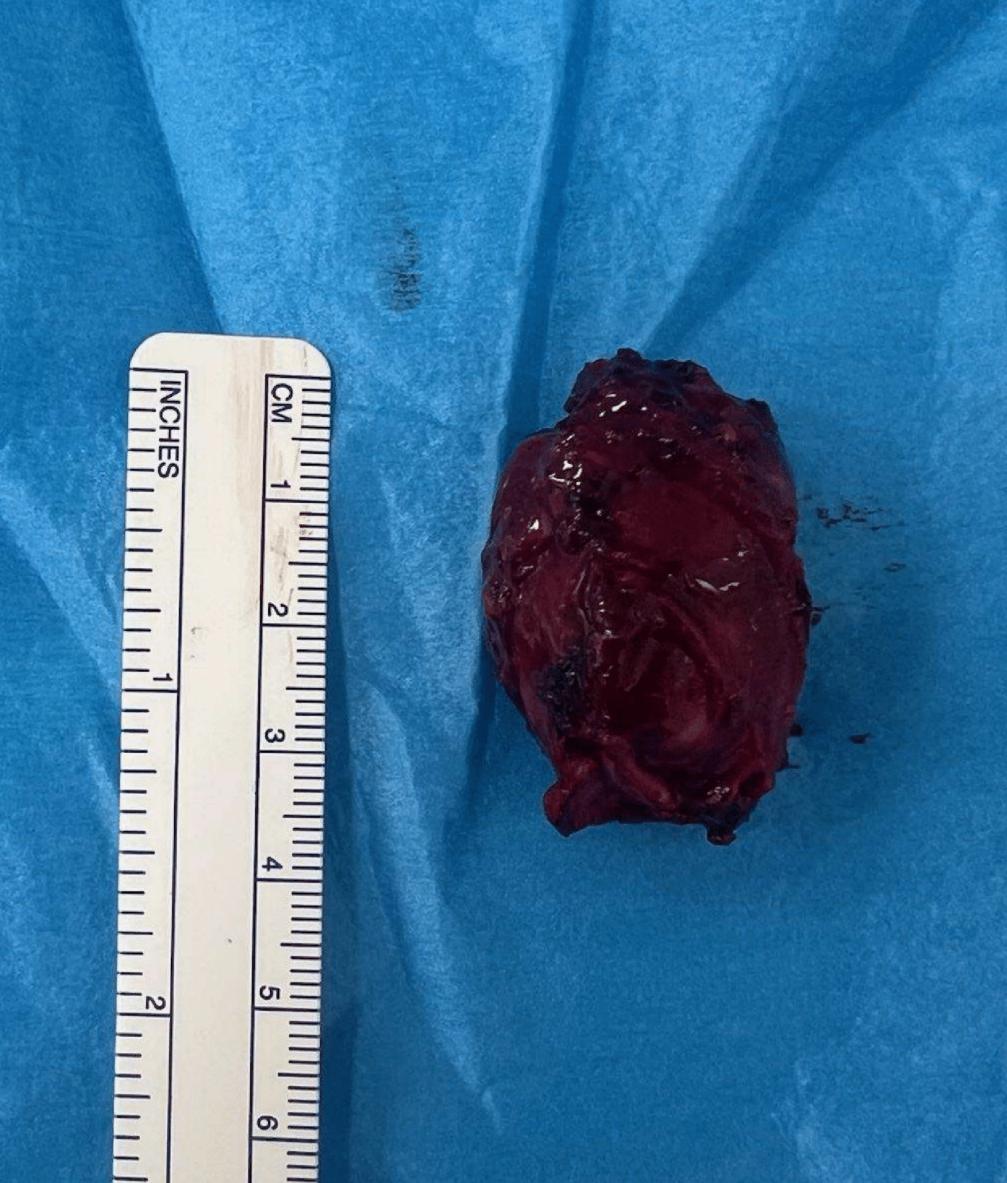
Photograph of the excised paraganglioma.

The excised tissues were sent for histopathological analysis, which confirmed the diagnosis of a sympathetic paraganglioma. Figure 6 showcases a photograph of the excised tumor.

## 5 HISTOPATHOLOGY

**FIGURE 7:**
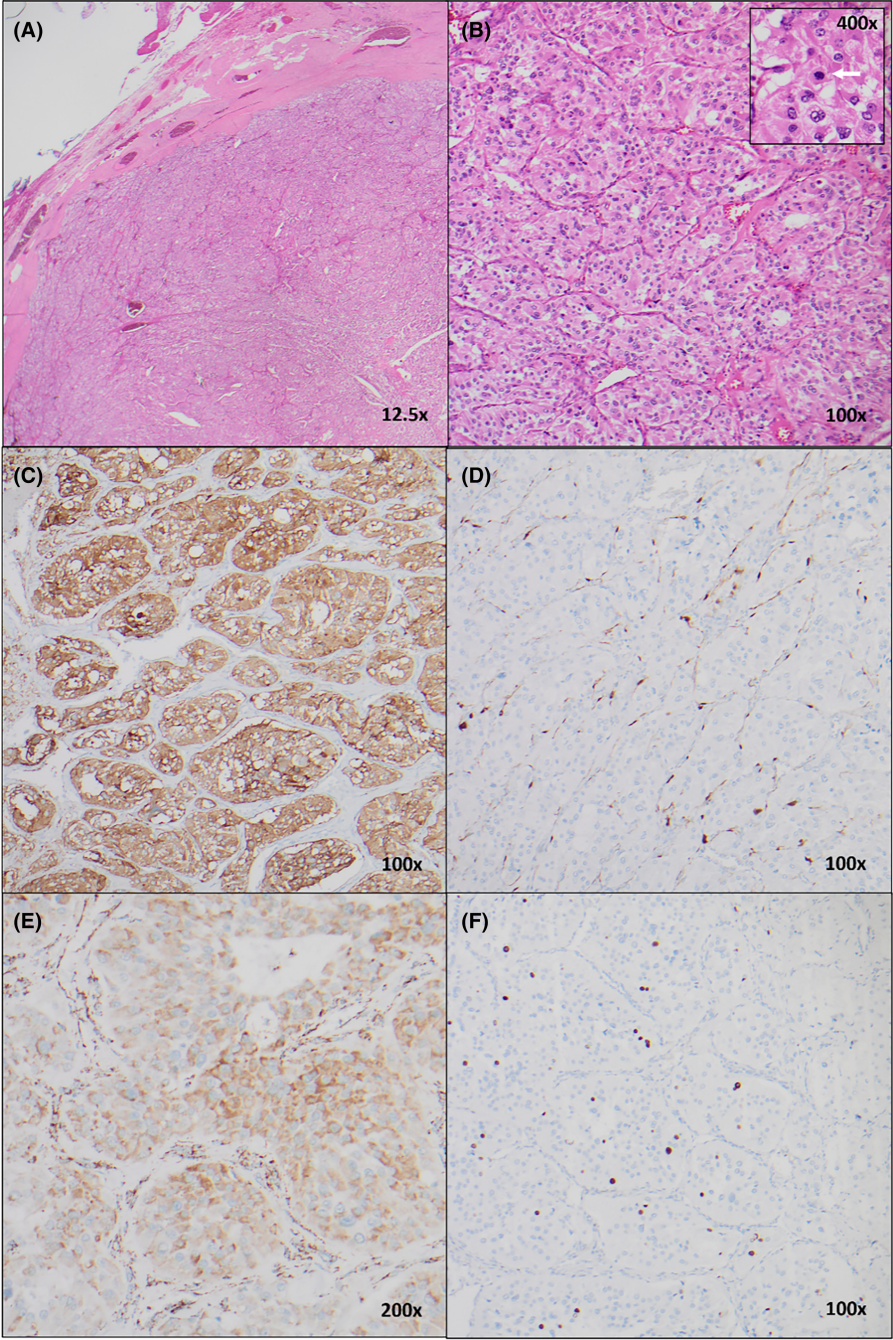
(A) Low power image showing well‐circumscribed peripheral edge of tumor with fibrous capsule (H&E, 12.5×); (B) Higher power image showing “zellballen” architecture characteristic of paraganglioma (H&E, 100×), with inset showing a mitotic figure (H&E, 400×); (C) Synaptophysin immunohistochemistry showing positive expression in tumor cells (100×); (D) S100 immunohistochemistry highlighting positive sustentacular cells at edges of nests (100×); (E) Succinate dehydrogenase B immunohistochemistry showing positive (retained) cytoplasmic expression (200×); (F) Ki67 proliferation index was up to 12% in the most active region (100×).

The specimen consisted of an ovoid tissue piece, 38 × 22 × 20 mm. Sectioning revealed an encapsulated, solid tumor with a homogenous tan cut surface. Microscopically, the tumor was well‐circumscribed, surrounded by a thick fibrous capsule (Figure 7A). It showed “Zellballen” architecture, composed of nests of epithelioid cells with eosinophilic, focally vacuolated cytoplasm and round to ovoid nuclei, separated by a thin network of capillaries and inconspicuous sustentacular cells (Figure 7B). Occasional mitoses were noted (Figure 7B, *inset*). Immunohistochemistry showed positive staining for synaptophysin (Figure 7C) and chromogranin (not shown), with positive immunostaining for S100 in sustentacular cells (Figure 7D). Succinate dehydrogenase B showed positive/retained cytoplasmic expression (Figure 7E). The Ki67 proliferation index was up to 12% in hotspots (Figure 7F). The surgical margins were negative.

## 6 POST‐OPERATIVE COURSE

After the surgery, the patient was admitted to the Intensive Care Unit for close observation. Despite the critical postoperative period, he remained hemodynamically stable without the need for vasopressor support or ventilation. Postoperative pain was managed through patient‐controlled analgesia (PCA) using oxycodone with bolus doses of 0.5–2 mg, a regimen that was maintained for 2 days. To mitigate thromboembolic risks, especially considering the partial resection of the iliac vein, prophylactic Enoxaparin was introduced and escalated to therapeutic doses by the next day.

Given the patient's stable condition, he was transferred to a general ward on the first postoperative day. Pre‐admission antihypertensives were gradually tapered and eventually ceased. The patient progressed well initially, tolerating a full diet, mobilizing independently, and passing flatus by the second day, albeit without bowel movements.

On Day 3 postoperatively, he began experiencing increased discomfort, marked by acute lower back pain, nausea, vomiting, extreme lethargy, and reduced appetite. These symptoms worsened over the following 2 days, prompting a reintroduction of PCA and starting IV hydration. By the fifth day, his pain intensified, accompanied by periodic fevers peaking at 38.5°C, with serum tests showing a drop in hemoglobin and rising inflammatory markers (Table 2). Despite moderate abdominal distension, there was no peritonitis, and bowel movements were notably absent. The surgical incision site remained intact, displaying no apparent signs of infection or bleeding. A septic screen, including urine MCS, blood cultures, and a chest X‐ray, yielded normal results.

**TABLE 2:** A sequential analysis of daily blood parameters in the postoperative period over 6 days, highlighting variations in hemoglobin, WBC, neutrophils, and CRP.

Parameter	Day 1	Day 2	Day 3	Day 5^a^	Day 6	Reference range
Hemoglobin	140	144	134 ↓	110 ↓	102 ↓	135–189 g/L
Hematocrit	0.41	0.42	0.38 ↓	0.32 ↓	0.30 ↓	0.40–0.53 L/L
WBC	15.1 ↑	11.4 ↑	9.2	11.9 ↑	11.5 ↑	4.0–11.0 × 10^9^/L
Neutrophils	13.14 ↑	8.21 ↑	6.07	9.28 ↑	8.51 ↑	1.80–7.50 × 10^9^/L
CRP	37.1 ↑	‐	87.9 ↑	150.0 ↑	237.9 ↑	<6.0 mg/L
Urea	3.4	3.6	2.9 ↓	2.9 ↓	3.4	3.4–9.0 mmol/L
Creatinine	62	65	57 ↓	58 ↓	63	60–110 umol/L
eGFR	>90	>90	>90	>90	>90	> = 90 mL/min/1.73 m^2^
Sodium	136	141	139	137	137	135–145 mmol/L
Potassium	4.2	4.0	4.0	4.0	3.8	3.5–5.2 mmol/L
Corrected Calcium	2.18	2.19	‐	2.26	2.26	2.10–2.60 mmol/L
Magnesium	0.91	0.75	‐	0.74	0.74	0.70–1.10 mmol/L
Phosphate	1.46	0.97	‐	1.24	0.99	0.75–1.50 mmol/L

^a^ No blood tests were conducted on the fourth postoperative day.

**FIGURE 8:**
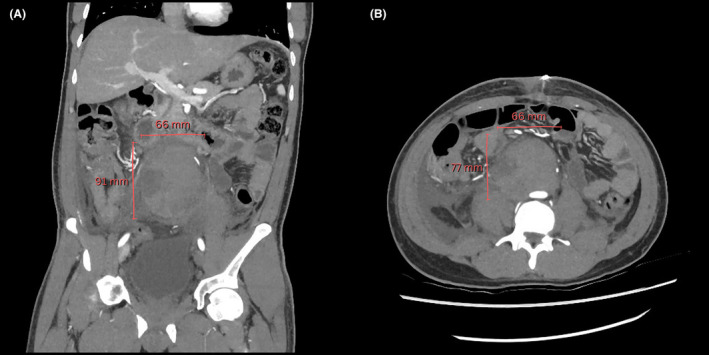
Coronal (A) and axial (B) abdominal CT angiography images showing the large non‐enhancing retroperitoneal hematoma (91 × 66 × 77 mm), extending from the common iliac bifurcations to the level of the inferior mesenteric artery at L3. The collection shows no contrast enhancement.

**FIGURE 9:**
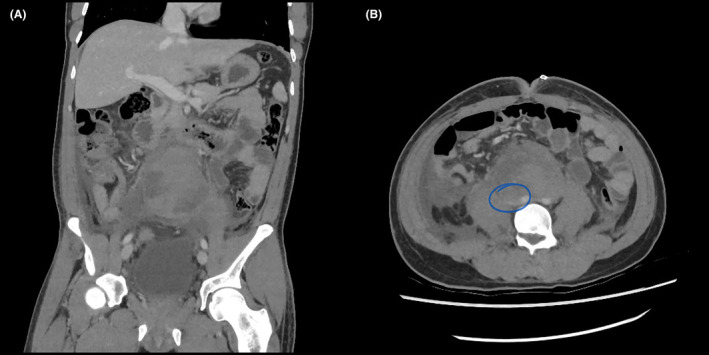
Coronal (A) and axial (B) slices from the portal venous phase demonstrating compression of the infrarenal inferior vena cava (blue circle) with presence of free fluid in the upper abdomen and right flank, and presacral stranding.

A subsequent triple‐phase abdominal CT identified a large retroperitoneal collection consistent with a postoperative haematoma, compressing the infrarenal inferior vena cava (Figures 8 and 9). The origin of the haematoma was not definitively identified, but given the surgical interventions and the patient's anticoagulation, there were multiple potential sources.

**FIGURE 10:**
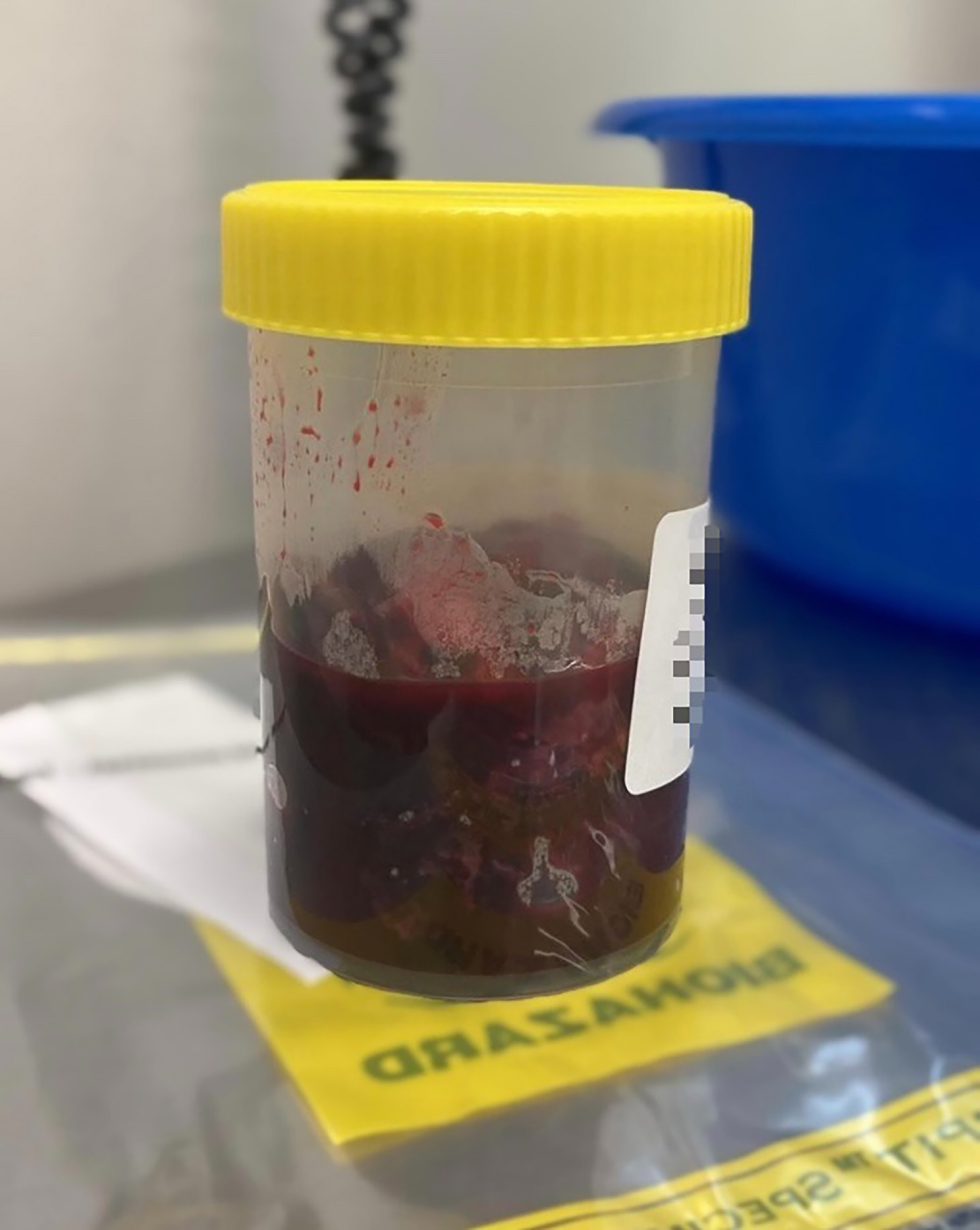
Image of the 100 mL evacuated haematoma from the retroperitoneum.

In light of these findings, a decision was made for a second surgery to evacuate the haematoma. The patient's enoxaparin was adjusted back to prophylactic dosing, and intravenous cefazolin was initiated. A week after the initial surgery, a second laparotomy was performed, resulting in the successful removal of a 100 mL clotted haematoma, with no intraoperative bleeding observed (Figure 10). The wound bed was thoroughly irrigated with saline, and a fibrin sealant patch was applied. MCS testing on the haematoma confirmed no microbial growth.

## 7 OUTCOME AND FOLLOW‐UP

Post the second surgery, the patient's symptoms markedly improved. By Day 2, he was on a full diet without further incidences of nausea or vomiting, and by the third day, regular bowel functions resumed. The patient's recovery trajectory was deemed optimal, evidenced by his independent mobility, the cessation of antibiotics by the fourth day, and the significant improvement in serum biomarkers (Table 3). He was discharged 5 days after the second surgery, totalling 11 days post initial laparotomy.

**TABLE 3:** Progression of the patient's hemoglobin levels, inflammatory markers, and other biochemical parameters following the second operation.

Parameter	Day 1	Day 2	Day 3	Day 4	Day 5	Reference range
Hemoglobin	99 ↓	98 ↓	105 ↓	103 ↓	115 ↓	135–189 g/L
Hematocrit	0.29 ↓	0.29 ↓	0.30 ↓	0.29 ↓	0.33 ↓	0.40–0.53 L/L
WBC	11.0	9.6	10.7	10.2	11.8 ↑	4.0–11.0 × 109/L
Neutrophils	8.25 ↑	6.53	7.92 ↑	7.34	8.26 ↑	1.80–7.50 × 109/L
CRP	263.7 ↑	309.3 ↑	257.1 ↑	174.4 ↑	122.4 ↑	<6.0 mg/L
Urea	3.7	2.2 ↓	2.9 ↓	2.1 ↓	2.3 ↓	3.4–9.0 mmol/L
Creatinine	47 ↓	52 ↓	53 ↓	53 ↓	59 ↓	60–110 umol/L
eGFR	>90	>90	>90	>90	>90	> = 90 mL/min/1.73 m^2^
Sodium	137	137	137	136	137	135–145 mmol/L
Potassium	4.2	3.9	3.8	3.9	4.8	3.5–5.2 mmol/L
Corrected Calcium	2.25	2.25	2.24	2.27	2.30	2.10–2.60 mmol/L
Magnesium	0.67 ↓	0.65 ↓	0.70	0.70	0.80	0.70–1.10 mmol/L
Phosphate	1.05	0.94	0.80	0.91	1.12	0.75–1.50 mmol/L

A comprehensive post‐discharge care plan was implemented, involving a coordinated multidisciplinary team. The patient's GP was tasked with overseeing wound care and monitoring blood pressure. Four weeks postdischarge, a re‐evaluation of plasma metanephrines was planned, the results of which would be discussed during an outpatient endocrinology clinic visit at 6 weeks. Given the potential hereditary predisposition for paraganglioma syndromes, consideration for genetic screening was warranted.

Six weeks postdischarge, a triple‐phase abdominal CT scan was organized to gauge postoperative progress and identify complications. A follow‐up vascular surgery clinic appointment was scheduled post‐scan to discuss the imaging findings and review the condition of the repaired left common iliac vein.

## 8 DISCUSSION

### 8.1 Understanding paragangliomas

Paragangliomas, rare neuroendocrine tumors arising from extra‐adrenal autonomic paraganglia, secrete catecholamines, commonly leading to hypertension, episodic headaches, and tachycardia.^1^ While paragangliomas share similarities with pheochromocytomas, their differentiation lies in their location and co‐occurrence patterns, predominantly developing in paravertebral ganglia across the thorax, abdomen, and pelvis.^2^

Up to 50% of paragangliomas are associated with inherited syndromes, including multiple endocrine neoplasia Types 2A and 2B, neurofibromatosis Type 1, and von Hippel Lindau, implicating various genes encoding the succinate dehydrogenase (SDH) enzyme complex.^3^, ^4^ Their epidemiology is nebulous due to their rarity, but the combined incidence with pheochromocytomas is roughly 0.8 per 100,000 person‐years.^5^ While the median age of diagnosis is in the late 40s, hereditary paragangliomas typically manifest earlier, often by the early 30s.^6^

Paragangliomas are sub‐classified based on origin: parasympathetic paragangliomas, primarily non‐secretory, are found in the neck and skull base along the glossopharyngeal and vagus nerves, while sympathetic variants, often catecholamine‐secreting, arise anywhere along the sympathetic chain.^7^ Notably, about 75% of sympathetic paragangliomas originate at the Organ of Zuckerkandl, a chromaffin cell‐rich structure near the aortic bifurcation and an embryological remnant of neural crest cells in some individuals.^8^ These frequently secrete catecholamines, producing symptoms mirroring adrenal pheochromocytomas, with approximately one‐fourth linked to a genetic syndrome.^9^

Diagnosis is a two‐step process that involves biochemical analysis of urinary and plasma fractionated metanephrines and catecholamines, followed by radiological imaging through CT, angiography, MRI, and PET.^10^, ^11^ The choice of PET radiopharmaceutical is contingent upon the specific pathogenic variants, as these directly influence the expression of receptors and metabolites in different paragangliomas. In this case, Gallium Ga‐68 DOTATATE was utilized ‐ a compound selectively absorbed by cells overexpressing the somatostatin receptor.^12^, ^13^

### 8.2 Diagnostic challenges

Paragangliomas, particularly those in the Organ of Zuckerkandl, present a formidable diagnostic challenge due to their rarity and often subtle manifestations. In younger patients without risk factors, treatment‐resistant hypertension can point towards atypical causes like paragangliomas, reinforcing the need for an exhaustive diagnostic workup.

The diagnosis employed a multi‐modal approach, leveraging serum normetanephrine biochemical testing and various imaging techniques essential for differentiating conditions like lymphadenopathy, adrenal masses, or other abdominal neoplasms. While contrast‐enhanced CT and 68Ga DOTATATE PET findings suggested a paraganglioma in the Organ of Zuckerkandl, the subsequent MRI was initially misinterpreted. This exemplifies the diagnostic complexities of paragangliomas and the imperative for vigilant radiological evaluation to avoid misdiagnosis.

The potential genetic implications of paragangliomas with inherited syndromes add another layer of complexity, even in the absence of a familial history. The immunohistochemical evidence of retained SDH B expression suggest that germline SDH mutations are not an aetiological risk factor in this case.

### 8.3 Multidisciplinary management

Emphasizing the significance of a multidisciplinary approach, this case involved collaboration among general practitioners, radiologists, endocrinologists, and vascular surgeons. Presented initially with blurred vision and discomfort without notable ocular anomalies, the patient was unexpectedly diagnosed with labile hypertension. Despite initial antihypertensive interventions, the underlying catecholamine‐producing tumor rendered them ineffective. Diagnostic clarity was achieved through a combination of radiological tools, including contrast‐enhanced CT, 68Ga DOTATATE PET/CT scan, and MRI.

Upon biochemical and radiological validation, the focus shifted to surgical management. The tumor's size and strategic location at the aortic bifurcation necessitated meticulous preoperative planning. Moreover, the potential catecholamine surge during surgery posed a risk of hypertensive crisis. Although abdominal paragangliomas can be addressed through open or endoscopic methods, factors such as tumor size, surgical anatomy, and indicators of local invasion often dictate the choice.^14^, ^15^, ^16^, ^17^ Endoscopic approaches are favored for extra‐adrenal paragangliomas due to reduced postoperative morbidity and shorter hospitalization.^15^, ^16^ However, large tumor size, challenging surgical anatomy, or radiographic and clinical indicators of local invasion may render an endoscopic approach impractical.^17^ The tumor's proximity to the aortic bifurcation in this case mandated an open procedure, acknowledging the potential for postoperative complications like retroperitoneal hematoma.

### 8.4 Recurrence and surveillance

Despite a <10% risk of local recurrence post‐total resection, it is crucial for healthcare practitioners to remain vigilant, especially in cases with multiple tumors, metastatic disease, familial paraganglioma, or extra‐adrenal tumors.^18^ Even ostensibly benign paragangliomas should not deter the implementation of annual life‐long follow‐ups, as 31% of patients in a series study spanning various regions showed persistent or recurrent disease, or the development of metachronous primary tumors.^19^

Following resection of catecholamine‐secreting paragangliomas, the National Comprehensive Cancer Network suggests repeat biochemical evaluation and imaging within 3 months.^20^ The benchmarks for successful operative treatment are normalized plasma or urinary levels of fractionated metanephrines and catecholamines, along with negative imaging results. Additionally, postoperative guidelines recommend monitoring blood pressure and biochemical markers semi‐annually for the first 3 years, then annually for the next decade, tailoring imaging studies to specific clinical indications.^20^

## 9 CONCLUSION

In reporting a young male with resistant hypertension and a subsequently diagnosed Organ of Zuckerkandl paraganglioma, this intricate case illuminates the diagnostic complexities and challenges inherent in managing such rare conditions. Emphasizing the crucial role of multi‐disciplinary collaboration, from GPs to vascular surgeons, the case report underscores the importance of comprehensive diagnostic evaluation, particularly when secondary hypertension arises in younger patients. Despite the successful intervention, postoperative complications highlighted areas for enhanced clinical decision‐making and the need for genetic considerations within paraganglioma management. Moving forward, refining diagnostic criteria, advancing surgical practices, and delving deeper into genetic predispositions are paramount in optimizing patient outcomes. Encouraging dedicated research on these fronts will not only bolster evidence‐based practices but also elevate the comprehensive care and management of paragangliomas.

## AUTHOR CONTRIBUTIONS

**Reabal Najjar:** Conceptualization; data curation; formal analysis; investigation; methodology; visualization; writing – original draft; writing – review and editing. **Elizabeth Paver:** Resources; supervision; visualization; writing – review and editing. **Jonathan McGuane:** Resources; visualization; writing – review and editing. **Gert Frahm‐Jensen:** Conceptualization; investigation; project administration; resources; supervision; writing – review and editing.

## FUNDING INFORMATION

The authors declare no financial support.

## CONFLICT OF INTEREST STATEMENT

The authors declare no conflicts of interest.

## CONSENT

Written informed consent for the publication of this case report was obtained from the patient. A copy of the written consent can be made available for review if requested.

## Data Availability

The authors confirm that the data supporting the findings of this study are available within the article.
